# Corticospinal Excitability in Children with Congenital Hemiparesis

**DOI:** 10.3390/brainsci6040049

**Published:** 2016-10-20

**Authors:** Chao-Ying Chen, Tonya L. Rich, Jessica M. Cassidy, Bernadette T. Gillick

**Affiliations:** 1 Department of Physical Medicine and Rehabilitation, College of Medicine, University of Minnesota, MMC 297, 420 Delaware St SE, Minneapolis, MN 55455, USA; chen4712@umn.edu (C.-Y.C.); rich1038@umn.edu (T.L.R.); 2 Department of Neurology, University of California, Irvine, Hewitt Hall, Room 1331, Irvine, CA 92697, USA; jmkream@gmail.com

**Keywords:** transcranial magnetic stimulation, motor evoked potential, motor threshold, perinatal stroke, cerebral palsy

## Abstract

Transcranial magnetic stimulation (TMS) can be used as an assessment or intervention to evaluate or influence brain activity in children with hemiparetic cerebral palsy (CP) commonly caused by perinatal stroke. This communication report analyzed data from two clinical trials using TMS to assess corticospinal excitability in children and young adults with hemiparetic CP. The results of this communication revealed a higher probability of finding a motor evoked potential (MEP) on the non-lesioned hemisphere compared to the lesioned hemisphere (*p* = 0.005). The resting motor threshold (RMT) was lower on the non-lesioned hemisphere than the lesioned hemisphere (*p* = 0.013). There was a significantly negative correlation between age and RMT (*r_s_* = −0.65, *p* = 0.003). This communication provides information regarding MEP responses, motor thresholds (MTs) and the association with age during TMS assessment in children with hemiparetic CP. Such findings contribute to the development of future pediatric studies in neuroplasticity and neuromodulation to influence motor function and recovery after perinatal stroke.

## 1. Introduction

Perinatal stroke is estimated to occur in as many as 1 in 2300 live births [1], and is the leading cause of cerebral palsy (CP) in children [2]. Stroke may occur during in-utero development and surrounding birth. Children with hemiparetic CP clinically display greater impairments on the side of body contralateral to stroke. The motor deficits, especially in the upper extremities, are attributed to an interrupted corticospinal pathway and subsequent brain organization [3,4,5]. Movement patterns as a result of hemiparetic CP may include mirroring that affects functional performance [6]. For example, when a child with hemiparetic CP is grasping an object using one hand, the other hand may move in the same manner simultaneously without isolated control.

Non-invasive brain stimulation (NIBS) techniques, specifically transcranial magnetic brain stimulation (TMS), may be used to understand brain excitability in adults and children with and without neurologic conditions. In adults with stroke, TMS has been widely researched to assess or influence corticospinal excitability and reorganization [7,8,9,10]. In children with CP, NIBS can also be used for corticospinal excitability assessment [11,12,13,14,15,16,17], as well as a neuromodulatory intervention, to improve motor outcomes. Two such types of interventions include repetitive TMS (rTMS) and transcranial direct current stimulation (tDCS) [18,19,20,21,22,23,24]. Despite the strong translational potential of NIBS for pediatric applications, the role of TMS in examining the brain reorganization and plasticity has been more thoroughly explored in adults. One reason may be due to the challenges of performing TMS assessment or intervention in children.

Successful TMS assessment and data collection requires a consistent neurophysiological response as referred to as a motor evoked potential (MEP) that investigators record using surface electromyography (EMG). TMS-elicited MEP responses may be variable in children with perinatal stroke for several neurodevelopmental and physiological reasons, such as age and heterogeneous characteristics of brain injuries. For example, corticospinal tract maturation and myelination continue to develop until adulthood [25], wherein an immature system may require higher TMS stimulator output to elicit an equivalent MEP in children compared to adults. In these cases, applying a supra-threshold stimulation intensity to investigate cortical mechanisms, which is the common TMS testing protocol, may not therefore be feasible due to maximum stimulator output (MSO) and the tolerance of the child to the procedure. Considering the gap in the neurophysiologic pediatric literature surrounding TMS studies in children with hemiparetic CP, the purpose of this communication is to analyze both MEP responses and motor threshold during initial/baseline TMS testing sessions from two clinical trials. We hypothesized that we would fnd (1) a higher level of corticospinal excitability on the non-lesioned hemisphere compared to the lesioned hemisphere and (2) decreased motor thresholds with increasing age of individual participants.

## 2. Materials and Methods

This communication summarizes the TMS testing results of two clinical trials from our pediatric research laboratory [18,26]. The TMS testing protocol for both studies involved assessment of the motor cortex hotspot that contributes to movement of targeted hand muscles (BiStim2, Magstim, Whitland, UK). We defined the hotspot as the location on the scalp that represents the underlying cortical response requiring the lowest TMS stimulator output to elicit an MEP in targeted hand muscles as recorded by EMG. We defined MEP as the peak-to-peak EMG amplitude equal or greater than 50 μV in at least 3 out of 5 trials. Motor threshold (MT) was determined as the lowest intensity of TMS stimulator output that elicited an MEP at the hotspot. We attempted to determine the participant’s MT with the hand muscles at rest (e.g., resting motor threshold, RMT). If an RMT was not found, we determined the participant’s active motor threshold (AMT). Due to variable challenges with maintaining isometric contraction in children with CP (i.e., cognitive understanding of the task, weakness or spasticity in the targeted muscle, and visual ability to see the EMG screen), when testing AMT, participants were asked to perform their own maximum muscle contraction instead of a pre-defined, sub-maximum contraction. If participants relaxed the muscle contraction due to misunderstanding of the task or fatigue before delivering TMS pulses, we repeated that specific trial. The AMT was determined when the TMS triggered EMG activity could be distinguished from background EMG activity at a level of ≥50 µV. General pediatric participant inclusion criteria for receiving TMS assessment and testing parameters in each study are summarized below.

### 2.1. Study 1: Primed Low-Frequency Repetitive Transcranial Magnetic Stimulation and Constraint-Induced Movement Therapy in Pediatric Hemiparesis: A Randomized Trial (rTMS Study) (ClinicalTrials.gov # NCT01104064) [18]

All subjects gave their informed consent for inclusion before they participated in the study. The study was conducted in accordance with the Declaration of Helsinki, and the protocol was approved by the Institutional Review Board (IRB) (IRB Code Number: 0910M72992; Approval Date: 19 November 2009).

The inclusion criteria for this study were children between 8 and 17 years of age who had congenital hemiparesis caused by perinatal stroke or periventricular leukomalacia. Movement criteria for the paretic hand required at least 10 degrees of active finger movements. Exclusion criteria included a history of seizures in the past two years, use of centrally activating medications (e.g., seizure or attention deficit medications), and/or use of botulinum toxin or phenol blocks in the past six months.

The MT on the lesioned hemisphere for each participant was evaluated through monitoring EMG of the extensor digitorum muscle. Based on previously published procedures using the 70 mm figure-of-eight TMS coil, we identified the area of the ’hand knob’ within the precentral gyrus of the frontal lobe [18]. Using individual head measurements and cortical excitability measures, we then were able to target the ‘hotspot’ or location that controls the hand muscle and identify the MT at this location [18]. The protocol allowed reaching a 100% MSO in TMS assessment. All participants were required to demonstrate an MEP on the lesioned hemisphere as a criterion to be enrolled to receive intervention in this study. Requiring an MEP on the lesioned hemisphere allowed for a more homogenous sample of participants. Thirty children initially qualified for the TMS assessment. However, 11 of the enrolled children were later excluded before intervention randomization due to lack of MEP obtained from the lesioned hemisphere.

### 2.2. Study 2: Synergistic Effect of Combined Transcranial Direct Current Stimulation/Constraint-Induced Movement Therapy in Children and Young Adults with Hemiparesis (tDCS Study) (ClinicalTrials.gov # NCT02250092) [26]

All subjects gave their informed consent for inclusion before they participated in the study. The study was conducted in accordance with the Declaration of Helsinki, and the protocol was approved by the Institutional Review Board (IRB) (IRB Code Number: 1408M53169; Approval Date: 11 September 2014).

This study is ongoing, yet all baseline TMS testing sessions are completed. The inclusion criteria for this tDCS study were children or young adults between 8 and 21 years of age who had congenital hemiparesis caused by perinatal stroke or periventricular leukomalacia. Exclusion criteria included a history of seizures in the past two years and use of botulinum toxin or phenol blocks in the past six months. Children on centrally-activating medications were included and dosaging noted.

The MT of the hotspot on both hemispheres for each participant was assessed through monitoring EMG in the same manner as the previous study; however, the muscle of interest was the first dorsal interosseous [26]. In this study, we used the frameless stereotactic neuronavigation system (Brainsight, Rogue Research, Montreal, QC, Canada) to guide the hotspot searching and MT identification by projecting each individual’s structural magnetic resonance imaging (MRI)—T1 to the system. The maximum intensity for single-pulse TMS testing in this study protocol was 85% of the MSO to enhance safety and tolerability during TMS assessment. This study required an MEP response on the non-lesioned hemisphere only. The absence of a MEP on the lesioned hemisphere did not result in exclusion. Although this broader enrollment criterion of including individuals with only contralesional MEP responses created a more heterogeneous sample, this allowed for inclusion of varying types of brain reorganization patterns. Twenty children qualified for the TMS assessment after the pre-enrollment screening and all of them met the criteria for the further intervention.

### 2.3. Data Management

TMS data from both the studies was used to compute the probability of locating consistent MEP responses and determining the MTs. Given that the TMS testing protocols differed as previously discussed, this report reflects (1) MEP responses on the lesioned hemisphere using the rTMS and tDCS study and (2) MEP responses on the non-lesioned hemisphere using the tDCS study only. Due to participants’ tolerance of the duration of the testing procedure in the tDCS study, (i.e., sleepiness, distractibility, inability to sit for an entire session, disinterest in the task), the AMT was not comprehensively assessed in the majority of participants who did not show an RMT on the lesioned hemisphere. Therefore, only the RMT data and the association with age of the lesioned hemisphere was analyzed in this communication. One child participated in both the rTMS (2009) and tDCS studies (2016). This specific participant displayed an AMT on the lesioned hemisphere in the rTMS study and displayed an RMT on the non-lesioned hemisphere in the tDCS study. These findings indicate that the participant’s corticospinal excitability status changed between the two studies. The data from both of the studies were included for further analysis. Demographic data of participants from both the tDCS and rTMS study are listed in Table 1.

### 2.4. Statistical Analysis

IBM SPSS Statistics 21 (IBM Corp, Armonk, NY, USA) was used for statistical analyses. A Chi-squared test was used to compare the probability of the presence of RMT on lesioned and non-lesioned hemispheres using tDCS study data. A Chi-square test was also used to compare the presence of RMT on lesioned hemispheres between rTMS and tDCS studies.

Using tDCS study data, Wilcoxon signed rank test and Spearman’s rank correlation coefficient were used to compare RMT values and examine the correlation between lesioned and non-lesioned hemispheres from participants who showed RMT on both hemispheres.

Spearman’s rank correlation coefficients were also used to investigate the relationship between age and RMT values on non-lesioned hemispheres in the tDCS study and between age and RMT values on lesioned hemispheres by combining rTMS and tDCS studies. A *p*-value less than 0.05 was considered as significant difference in all statistical analyses.

## 3. Results

### 3.1. Presence of MEP on the Lesioned and Non-Lesioned Hemispheres in Both the rTMS and tDCS Studies

In the rTMS study, 30 children initially participated in the TMS assessment and 19 of them showed MEPs on the lesioned hemisphere. Among these 19 remaining participants, 10 children revealed an RMT and the other nine children showed an AMT.

**Figure 1:**
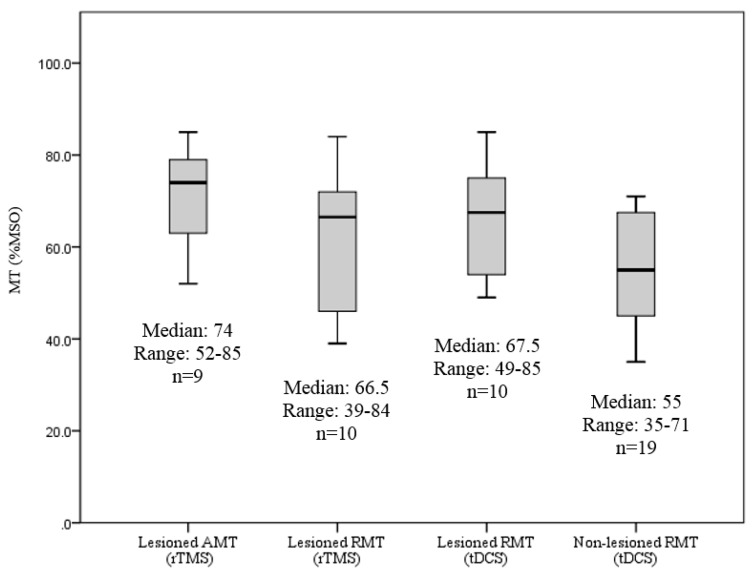
Boxplot reflecting medians and ranges of RMTs in both the rTMS (lesioned hemisphere) and tDCS (lesioned and non-lesioned hemispheres) studies. RMT: Resting motor threshold; rTMS: Repetitive transcranial magnetic stimulation; tDCS: Transcranial direct current stimulation; %MSO: Percentage of maximum of machine output.

In the tDCS study, 20 children or young adults participated in the TMS assessment and MEPs, determined either by RMT or AMT, were obtained in the full sample on the non-lesioned hemisphere. On the lesioned hemisphere, 10 children displayed an RMT. The remaining 10 children showed an absent MEP, an AMT, or were not able to tolerate the assessment during the hotspot determination. On the non-lesioned hemisphere, 19 children demonstrated an RMT and one child displayed an AMT. The Table 2 summarizes MEP presentation across the studies and Figure 1 lists MT values.

The Chi-square tests indicated a significant relationship between the presence of an RMT and hemisphere (lesioned vs. non-lesioned) in the tDCS study (X^2^ (1, *N* = 40) = 8.03, *p* = 0.005). There was no relationship between the presence of an RMT on the lesioned hemisphere between studies (rTMS vs. tDCS) (X^2^ (1, *N* = 50) = 0.78, *p* = 0.377).

### 3.2. RMT Values between Lesioned and Non-Lesioned Hemispheres

**Figure 2:**
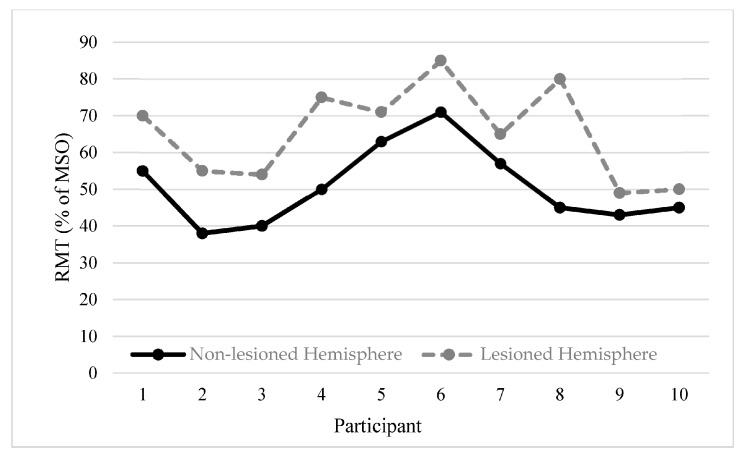
RMTs of lesioned (grey dashed lines) and non-lesioned (black solid lines) hemisphere. Individual RMTs of both the lesioned and non-lesioned hemispheres in 10 participants in the tDCS study. A significant correlation between lesioned and non-lesioned sides RMTs was found (*r_s_* = −0.64, *p* = 0.044).

A comparison of the RMT values between hemispheres was performed for participants in the tDCS study. Ten children demonstrated an RMT on both the lesioned and non-lesioned hemispheres. The Wilcoxon signed rank test showed a significant difference (Z = −2.49, *p* = 0.013) of the RMT values between the lesioned and non-lesioned hemisphere. Spearman’s rank correlation coefficient showed significantly strong correlation between lesioned and non-lesioned hemispheres (*r*_s_ = 0.64, *p* = 0.044) (Figure 2).

### 3.3. Influence of Age on RMT

**Figure 3:**
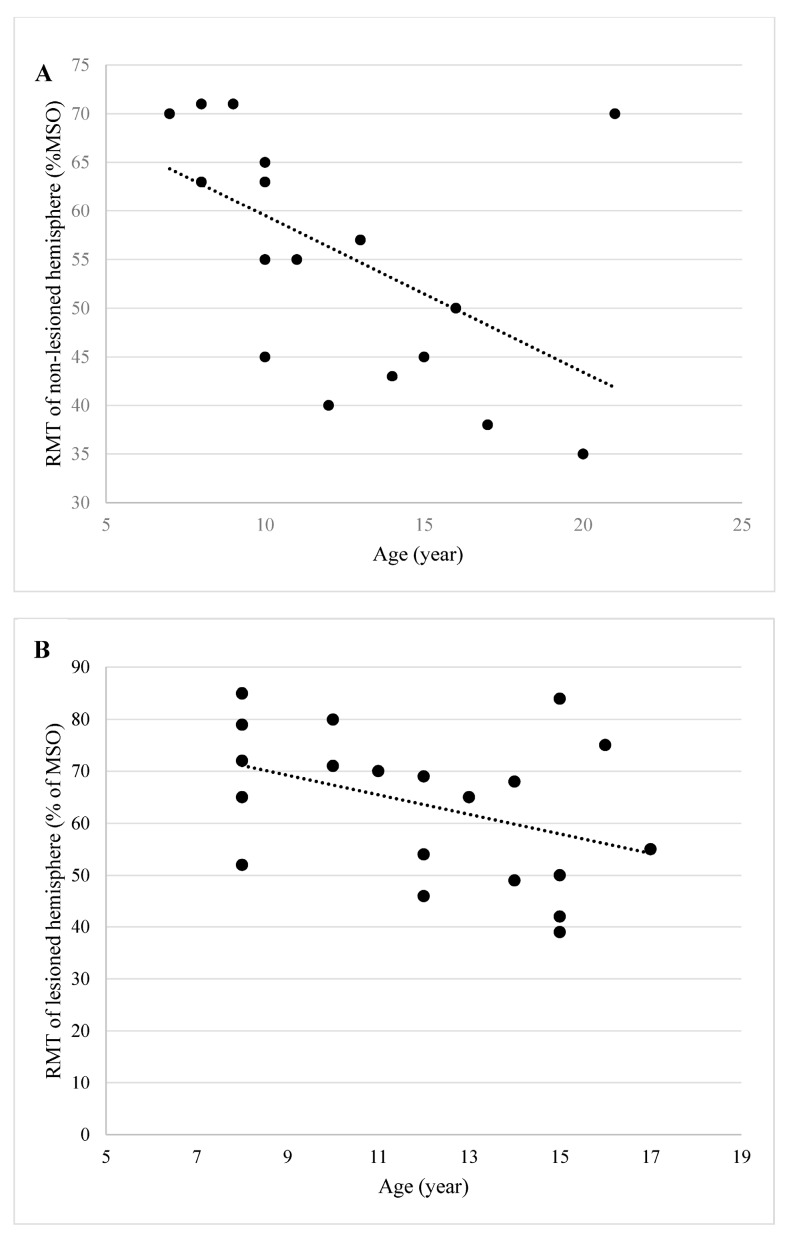
Linear correlation between individual participant’s age and RMT values of the non-lesioned and lesioned hemispheres. (**A**) Strong negative correlation between RMTs of the non-lesioned hemisphere and age (*r_s_* = −0.65, *p* = 0.003); (**B**) No correlation between RMTs of the lesioned hemisphere and age (*r_s_* = −0.37, *p* = 0.105).

In the tDCS study, 19 of 20 (95.0%) children demonstrated an RMT on the non-lesioned hemisphere. The Spearman signed rank test showed a significantly strong negative correlation (*r_s_* = −0.65, *p* = 0.003) between RMT and age (Figure 3A). By combining the data from both the rTMS and tDCS studies, there were 20 children who demonstrated an RMT on the lesioned hemisphere. The Spearman singed rank test did not show a significant correlation (*r_s_* = −0.37, *p* = 0.105) between RMT and age (Figure 3B).

## 4. Discussion

This purpose of this communication is to summarize the corticospinal excitability characteristics from two studies, using TMS in children and young adults with congenital hemiparesis. Application of TMS to find the brain location that controls a target muscle is an initial component of the study design in NIBS studies. Therefore, the presence of an MEP is often a critical inclusion criterion. Determining the presence of an MEP response on the lesioned hemisphere presents greater challenges due to factors such as lesion location and extent. In children, the immaturity of the corticospinal tract also poses additional difficulty. The findings in this communication support these arguments.

Consistent with our first hypothesis, across both studies (rTMS and tDCS) there was a higher probability of finding an RMT from the non-lesioned hemisphere with lower RMT values than the lesioned hemisphere. Based on the tDCS study, the probability of identifying an RMT on the non-lesioned hemisphere was approximately two times greater than the lesioned hemisphere. If a study protocol requires identifying an RMT from the lesioned hemisphere, the estimated sample size for recruitment needs to account for participants who do not demonstrate MEP responses from the lesioned hemisphere. In addition, a study protocol that utilizes either RMT or AMT as a criterion will have a higher probability of enrollment. We observed a 33% increase in success rate of finding an MEP from the lesioned hemisphere in the rTMS study by using both RMT and AMT. However, in participants with impaired neuromotor function who have difficulty maintaining a consistent isometric muscle contraction during AMT testing, AMT may be more appropriate for a general search for ‘excitable’ brain locations but not for other comparative TMS testing parameters, such as AMT values and peak-to-peak amplitude of MEP. We did not find significant differences on the probability of detecting an RMT on lesioned hemisphere between the rTMS and tDCS studies. Therefore, we cannot conclude if different population inclusion criteria and methodologies, such as the use of a neuronavigation system to guide hotspot searching, influences the probability of finding RMT.

In both studies, we observed an overall higher RMT from the lesioned hemisphere. Supra-threshold stimulation intensities are used frequently for assessing cortical excitatory and inhibitory mechanism using single-pulse and paired-pulsed TMS measurements [27,28]. The pediatric population may not tolerate these measurements due to high stimulation intensity. Further, these measurements are not feasible when a supra-threshold value is higher than 100% MSO (i.e., 110% or 120% RMT). Although the RMTs were higher on the lesioned hemisphere, there was a strong correlation between lesioned and non-lesioned hemispheres. For example, with a participant who has less excitable brain, the percentage of MSO needed to elicit an MEP may then be high on both hemispheres. This result indicates that even with early brain injuries, individual variability remains a key factor influencing the cortical excitability tested by TMS in children with congenital hemiparesis.

Similar to previous studies reporting significant correlations between age and RMT in children with typical development and on the non-lesioned hemisphere in children with CP [27,29], we also found a significant association between RMT on the non-lesioned hemisphere and participant age. As reported in previous studies, the correlation between MT values and age may be attributed to the changes of corticospinal tract through development in infants and degeneration of the tract in older adults [30,31]. We hypothesized that a higher RMT would be detected in studies investigating younger children. In this case, the choice of TMS testing parameters during construction of the study design should then be considered. In contrast to the non-lesioned hemisphere, we did not find a significant correlation between age and RMT on the lesioned hemisphere. Ongoing development of corticospinal tract, however involved in the neurologic injury, may contribute to the asymmetries discovered in this analysis [11].

A limitation of this communication is that the characteristics of our study populations, such as the history of seizure and the use of medication, was narrowed during pre-TMS screening process based on the inclusion and exclusion criteria. Therefore, the estimated percentages of qualified sample sizes from the TMS testing may not be applied to studies with different criteria. Moreover, the tDCS study established the highest stimulator intensity for TMS assessment set at 85% MSO. The probability of finding an RMT on the lesioned hemisphere may be higher without this limit. Future research, to comprehensively summarize and compare existing evidence regarding factors that may influence corticospinal excitability characteristics, will provide broader and more accurate information to advance the research design in this area.

## 5. Conclusions

This report summarizes MEP responses and RMT values in children of different ages and provides preliminary evidence to guide future TMS study design in children with hemiparetic CP due to perinatal stroke. Planned sample sizes may need to be larger when examining corticospinal excitability on the lesioned hemisphere than testing on the non-lesioned hemisphere in children with hemiparetic CP. In addition, the TMS testing parameters or intended neuromodulatory intervention must consider the potential for higher RMT values on the lesioned hemisphere, especially in younger children. Consideration of the neurophysiological responses during TMS assessment, such as the presence of an MEP on both hemispheres and the child’s potential to exhibit a high motor threshold, may aid in designing feasible and sufficiently powered study protocols that ultimately enhance functional outcomes in children with motor impairments caused by early brain injuries.

## Figures and Tables

**Table 1:** Demographic data of enrolled participants in the rTMS and tDCS studies.

-	rTMS (*n* = 30)	tDCS (*n* = 20)
Sex (Male:Female)	18:12	9:11
Age (Mean (range))	10.4 (7–16)	12.2 (7–21)
Lesioned Hemisphere (Right:Left)	10:20	5:15
MACS levels	I, II and III ^a^	I, II, III and IV

^a^ Only 19 participants who showed MEP of the lesioned hemisphere and enrolled in the rTMS study had MACS data. Abbreviation: rTMS: repetitive transcranial magnetic stimulation; tDCS: transcranial direct current stimulation; MACS: Manual Ability Classification System.

**Table 2:** Summary of the presence of an MEP in the rTMS and tDCS studies.

Hemispheres	rTMS (*n* = 30)		tDCS (*n* = 20)	
	Lesioned	Non-lesioned	Lesioned	Non-Lesioned
*N* of MEP presence (%)	19 (63.3%)	NA	12 (60.0%)	20 (100.0%)
*N* of RMT presence (%)	10 (33.3%)	NA	10 (50.0%)	19 (95.0%) *
*N* of AMT presence (%)	9 (30.0%)	NA	2 (10.0%)	1 (5.0%)

* Significant relationship between the presence of RMT and the side of hemisphere in the tDCS study (*p* = 0.005). Abbreviation: rTMS: repetitive transcranial magnetic stimulation; tDCS: transcranial direct current stimulation; MEP: motor evoked potential; RMT: resting motor threshold; AMT: active motor threshold; *N* = number of participants; NA: Not assessed.
